# Environmental recidivism in Sweden: distributional shape and effects of sanctions on duration of compliance

**DOI:** 10.1007/s11135-017-0493-8

**Published:** 2017-03-04

**Authors:** Gebrenegus Ghilagaber

**Affiliations:** Department of Statistics, Stockholm University, 106 91 Stockholm, Sweden

**Keywords:** Environmental regulations, Environmental sanctions charges, Time to re-offens, Modelling duration data, Recidivism, Sweden

## Abstract

The study examines the association between the size of previous environmental sanction charges and subsequent compliance towards environmental regulations. Data used for the study come from about 9000 Swedish firms fined sometime between January 2002 and December 2012. Probabilities of compliance across various levels of sanctions are estimated using life-table methods and tested for equality using standard nonparametric methods. Association between size of sanction charges and subsequent behaviour is modelled by proportional hazard model for the rate of recidivism as well as by a family of flexible parametric accelerated failure-time models for the duration of compliance. The results show that duration of compliance may be described by a log-normal distribution. Further, it is demonstrated that sanctions charges do have significant detering effects on the risk of recidivism though the strength of the detering effect depends on whether or not we account for other possible correlates of recidivism. Possible explanations of the results and their policy implications are discussed; limitations of the current study highlighted; and potential extensions for future studies outlined.

## Introduction

The overall aim of this paper is to measure the effect of environmental sanction charges on subsequent behavior with regard to violation to environmental regulations. Effect, in turn, is defined as a change that has occurred as a result of a specific measure taken—that otherwise would not have occurred or occurred at a latter time. It is then clear that it is not an easy task to measure effect as there are many variables which may influence environmental behavior of firms and individuals and the state of the environment, irrespective of enforcement actions. Further, substantial time may elapse between the application of enforcement measures and changes made evident in the environment.

National and international environmental agencies use a wide range of indicators to assess environmental conditions in general and the efficiency of enforcement measures in particular. One such measure suggested has been the extent of recidivism—the act of repeating violation to environmental regulations after a firm has been fined (penalized) for that behavior. Rates of recidivism and the duration in compliance have been suggested as output measures (International Network for Environmental Compliance and Enforcement 2008). Potential flaws in using recidivism ratios as measures of regulatory efficiency is outlined in a report by U.S. Environmental Protection Agency—Office of Enforcement and Compliance Assurance (2008). The main concern is that it is not possible to generalize observed recidivism rates among facilities which were inspected to those which were not inspected because some entities will be missed committing acts which, if they were caught to do so, would constitute recidivism. Because of this drawback, it is suggested to use a measure of chronic noncompliance as an alternative to recidivism rates. A potentially useful formulation of chronic recidivism suggested in the literature is the average or median length of time facilities/firms spend in compliance/noncompliance.

However, little is known about the empirics of environmental recidivism. In particular, to the best of our knowledge, there has not been any study proposing appropriate statistical methods to analyze data on length of compliance (time to recidivism) and model its association with background characteristics of facilities.

This paper attempts to fill this gap in the literature by presenting a number of statistical procedures of varying degree of complexity. The procedures are then illustrated using data on about 9000 Swedish firms which were fined sometime between January 2002 and December 2012. The goal of the study is to examine the effect of the size of sanction on the length of compliance.

In Sect. 2, we describe the data in more details. Section 3 presents a number of appropriate statistical methods and illustrates them empirically. These methods include Kaplan–Meier and Life Table methods for estimating survival functions; nonparametric Log-Rank and Breslow (Generalized Wilcoxon) tests for comparing the survival functions, Cox proportional hazard model for the rate of recidivism as well as a family of flexible parametric accelerated failure-time models for the duration of compliance. The last section ties up the contents of the paper in the form of concluding remarks, outline of limitations of the study, and potential extensions for further study.

## The data set

Since 1999, the Swedish Environmental Protection Agency has compiled statistics on imposed environmental sanction charges (Naturvårdsverket 2010). According to the source, Environmental sanction charges are administrative charges accruing to the government and they can be between SEK 1000 and SEK 1,000,000. Municipalities, county administrative boards and other central supervising authorities can decide that sanction charges be paid by those carrying out an activity, for which they have not been granted permission or which does not comply with the conditions given in the environmental code. We have got access to such data from January 2002 up to December 2012.

The initial data set consisted of 8983 cases (decisions on sanction charges) that took place sometime between January 2002 and December 2012 (see Table 1). Thus, of the 8983 cases/firms analyzed in this study, 4867 (54.18%) were fined less than 5000 SEK for previous offense, 3093 (34.43%) were fined 5000–10,000 SEK for previous offense while the rest 1023 (11.39%) were fined more than 10,000 SEK for previous offense.

**Table 1:** Distribution of recidivism and exposure across covariates

Covariate	Levels	# firms	Recid.	% Recid.	Exp.^a^	Rate^b^	RR^c^
Size of previous sanction (SEK)	<5000	4867	1928	39.61	173,416	11.12	2.55
	5000–10,000	3093	1080	34.92	139,903	7.72	1.77
	≥10,000	1023	271	26.49	62,086	4.36	1
Number of employees	None	2767	699	25.26	137,134	5.10	0.16
	1–9	1909	404	21.16	88,805	4.55	0.15
	10–99	2299	678	29.49	101,790	6.66	0.21
	≥100	2008	1498	74.60	47,676	31.42	1
Implementing auth.	Municpalities	7722	2888	37.40	321,687	8.98	1.69
	County admin	549	225	40.98	22,543	9.98	1.88
	Other auth.	712	166	23.31	31,175	5.32	1
Motive group^d^	Group 1	1827	543	29.72	94,593	5.74	1.06
	Group 2	2118	550	25.97	146,604	3.75	0.69
	Group 3	4672	2108	45.12	119,849	17.59	3.24
	Group 4	366	78	21.31	14,359	5.43	1
	Total	8983	3279	36.50	375,405	8.73	–

^a^Exp. stands for the accumulated period/months in which the firms were exposed to the risk of recidivism

^b^The Recidivsm Rates (Recid./Exp.) are initial estimates of the intensities when no account is taken for other factors. They are expressed per 1000 exposure months. A crude estimate of the overall recidivism rate is thus (3279/375,405) * 1000 = 8.73 re-offenses per month for every 1000 firms or, equivalently, about 105 re-offenses for every 1000 firms per year

^c^RR stands for Relative Rates (relative to that of the highest level of each covariate)

^d^ The motives were grouped as follows

Motive Group 1: Species protection provisions, waste, electrical and electronical products, chemical products and biotechnical organisms, unknown

Motive Group 2: Environmentally hazardous activity and public health safety

Motive Group 3: Environmental risk areas

Motive Group 4: Open-pit mining/source and agriculture, violations of provisions for gene technology

By the end of the follow-up period (31 December 2012), 3279 (36.50%) of the firms have committed first re-offense while the rest 5704 (63.50%) were still complying and, hence, considered as censored. Of the 3279 recidivists 1928 (58.80%) were fined less than 5000 SEK for previous offense, 1080 (32.94%) were fined 5000–10,000 SEK for previous offense while the rest 271 (8.26%) were fined more than 10,000 SEK for previous offense. The overall mean survival time was 76 months while the mean survival times for the three categories were 63, 79, and 91 months respectively.

A first impression we get from Table 1 is that while the percentage of recidivists coming from the 2nd group of firms is about the same as their percentage sizes in the entire sample, the percentage of recidivist firms from the 1st groups (those fined with less than 5000 SEK for previous offense), 58.80%, is higher than their percentage sizes in the entire sample (54.18%). On the other hand, the percentage of recidivists from the 3rd group (those fined with more than 10,000 SEK for previous offense), 8.26%, is less than the corresponding percentage contribution of these firms to the entire sample, (11.39%). In the next section, we shall present various analytical tools that can be used to formally test this differential in recidivism across levels of sanction charges.

## Modelling the association between sanction charges and recidivism

### Non-parametric estimation and comparison of survival functions

**Fig. 1:**
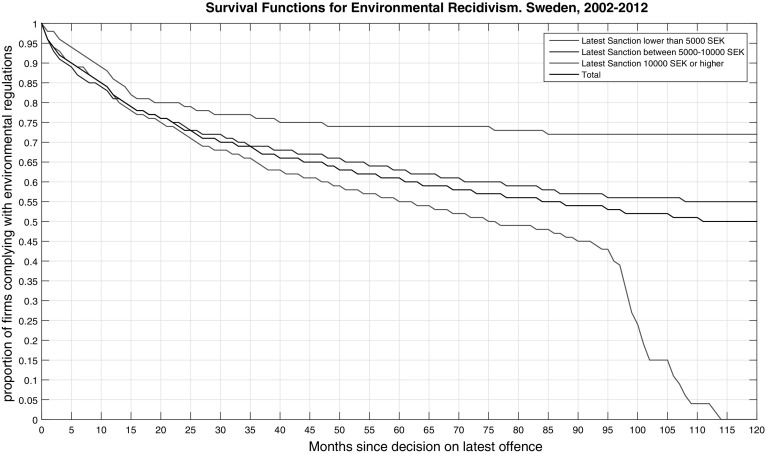
Proportion of firms complying with environmental regulation across size of latest sanctions charges

We begin with estimation and comparison of basic survival functions across the three levels of sanction charges. Life-table (actuarial) estimates of survival values are plotted in Fig. 1. As shown in the figure, the survival probabilities (probabilities of complying with environmental regulations), $S_{j}(t)$, by the end of the observation time are 0%, 55%, and 72% for the three groups of firms. In other words, by the end of the follow-up time (31 December 2012), 100% of the first group of firms (those who were charged less than 5000 SEK) have committed an environmental re-offense. The corresponding values for the 2nd and 3rd groups of firms was 45 and, 28%, respectively. The figure also shows that the 1st quartiles (the number of months by which 25% of the firms have committed re-offense) are 22 months for the entire sample, 20 months for the first group (those who were charged less than 5000 SEK), 23 months for the second group (those who were charged 5000–10,000 SEK) and 45 months for the third group (those who were charged 10,000 SEK or higher). The 2nd and 3rd quartiles for the 1st group of firms are 76 and 99 months, respectively while the 2nd and 3rd group of firms have not yet reached 50% of recidivism by the end of the observation time.

A formal test of the hypothesis:

$$H_{0}:S_{1}(t)=S_{2}(t)=S_{3}(t)$$

against

$$H_{1}:S_{i}(t)\ne S_{j}(t)\quad\text{for\,at\,least\,one\,pair}\,(i,j),$$

shows that there are significant differences between the survival functions of the different groups of firms. The Chi-square values are $\chi ^{2}=141$ (Log-Rank) and $\chi ^{2}=64$ (Generalized Wilcoxon) tests—both with *p* values less than 0.001. Further, pairwise-tests for equality of the survival curves yield the results displayed in Table 2 which, again, indicate statistically significant differences between the survival curves of the three groups of firms classified by size of previous sanctions charges.

**Table 2:** Results of tests for equality of survival functions

Test statistic	Null hypothesis	Chi-square	*p* value
Log-rank	${ S}_{1}{ (t)=S}_{2} { (t)}$	41.70	<0.001
	${ S}_{1}{ (t)=S}_{3}{ (t)}$	148.27	<0.001
	${ S}_{2}{ (t)=S}_{3}{ (t)}$	47.60	<0.001
Breslow (Generalized Wilcoxon)	${ S}_{1}{ (t)=S}_{2} { (t)}$	8.50	<0.001
	${ S}_{1}{ (t)=S }_{3}{ (t)}$	64.04	<0.001
	${ S}_{2}{ (t)=S}_{3}{ (t)}$	33.02	<0.001

### A logistic-regression model for the probability of recidivism

Let *Y* be an indicator of recidivism:

$$Y_{k}=\left\{ \begin{array}{ll} 1,&\quad\text{if\,firm}\,k\,\text{is\,a\,recidivist,\,i.e.\,has\,committed\,re-offense\,within\,the\,follow\,up\,period}\\ 0,&\quad\text{otherwise,\,i.e.\,if\,firm}\,k\,\text{was\,still\,complying\,until\,the\,end\,of\,the\,follow\,up\,period} \end{array} \right.$$

Let *p* = *P*(*Y* = 1) be the probability that a randomly selected firm is a recidivist. Our goal is to model this probability *p* as a function of the size of sanctions charge using a binary logistic regression model:

1 $$\log it(p)=\ln \left( \frac{p}{1-p}\right) =\beta _{0}+\beta _{1}X_{1}+\beta _{2}X_{2}$$

where $X_{1}$ is a dummy (0, 1) variable indicating firms from group 1, and $X_{2}$ is a dummy (0, 1) variable indicating firms from group 2. Firms from group 3 (those who were charged 10,000 SEK or higher) are used as reference levels, and both $X_{1}$ and $X_{2}$ have value 0 over all firms in group 3. The results (see Table 3) show a significant 49% higher odds of recidivism for firms in group 2 (those who were charged 5000–10,000 SEK) and even a highly significant 82% higher odds of recidivism for firms in group 1 (those who were charged less than 5000 SEK).

**Table 3:** Results from a logistic regression on the probability of recidivism

Sanctions (SEK)	${\widehat{\beta }}_{i}$	se(${\widehat{\beta }}_{i}$)	Wald-stat	*df*	*p* value	Odds ratio (OR)	95% CI for odds ratio
<5000	0.60	0.08	56.25	1	0.00	1.82	(1.57; 2.12)
5000–10,000	0.40	0.08	25.00	1	0.00	1.49	(1.27; 1.74)
Constant	−1.02	0.07	212.32	1	0.00		

The corresponding probabilities of recidivism for each group of sanctions charges may be computed as follows (Table 4).

**Table 4:** Probabilities of recidivism across size of sanction charges

	Sanction charges on previous offense (SEK)		
Recidivism indicator Y	<5000	5000–10,000	≥10,000
*Y* = 1 (Recidivism)	${ p}_{1} { =}\frac{\exp \left[ \beta _{0}+\beta _{1}\right] }{1+\exp \left[ \beta _{0}+\beta _{1}\right] }$	${ p}_{2} { =}\frac{\exp \left[ \beta _{0}+\beta _{2}\right] }{1+\exp \left[ \beta _{0}+\beta _{2}\right] }$	${ p}_{3} { =}\frac{\exp \left[ \beta _{0}\right] }{1+\exp \left[ \beta _{0} \right] }$
*Y* = 0 (Compliance)	${ 1-p} _{1}{ =}\frac{1}{1+\exp \left[ \beta _{0}+\beta _{1}\right] }$	${ 1-p}_{2}{ =}\frac{1}{1+\exp \left[ \beta _{0}+\beta _{2}\right] }$	${ 1-p}_{3}{ =}\frac{1}{1+\exp \left[ \beta _{0}\right] }$
Total	1	1	1

Substituting the corresponding estimates (${\widehat{\beta }}_{i}$ in Table 3) yields the following probabilities:

$${\widehat{p}}_{1}= \frac{\exp \left[ \widehat{\beta }_{0}+{\widehat{\beta }}_{1} \right] }{1+\exp \left[ \widehat{\beta }_{0}+{\widehat{\beta }}_{1}\right] }= \frac{\exp \left[ -1.02+0.60\right] }{1+\exp \left[ -1.02+0.60\right] } =0.40,\quad{\widehat{p}}_{2}= \frac{\exp \left[ \widehat{\beta }_{0}+{\widehat{\beta }}_{2} \right] }{1+\exp \left[ \widehat{\beta }_{0}+{\widehat{\beta }}_{2}\right] }= \frac{\exp \left[ -1.02+0.40\right] }{1+\exp \left[ -1.02+0.40\right] } =0.35,$$

and, lastly

$${\widehat{p}}_{3}=\frac{\exp \left[ {\widehat{\beta }}_{0}\right] }{1+\exp \left[ {\widehat{\beta }}_{0}\right] }=\frac{\exp \left[ -1.02\right] }{1+\exp \left[ -1.02\right] }=0.27.$$

Thus, firms in the the 3rd group (those which had highest sanction charges for previous offenses) are much less likely to commit recidivism compared to those in 1st and 2nd groups (those which had lower sanctions charges).

As shown above, the logistic regression model provides a general picture of the association between the probability of recidivism and the size of previous sanctions charges. However, it should be noted that the model is static in the sense that the focus is whether or not recidivism has occurred sometime within the follow-up period—with no account taken to the length of time until recidivism. In the next two subsections, we describe and implement two dynamic models that take into account the time until recidivism and, hence, use the data more efficiently. The Cox proportional hazards model is presented below while the last subsection presents the accelerated failure-time (AFT) models.

### Hazard models for the rate of recidivism

In analyzing recidivism data, interest may focus on examining the effects of sanctions on the hazard function of recidivism. Such a function, commonly denoted by $\lambda (t)$, is defined as the instantaneous rate at which recidivism occurs at a specific point *t*:

2 $$\lambda (t)=\lim _{\Delta t\longrightarrow 0}\frac{P\left[ t<T\leqslant t+\Delta t|T>t\right] }{\Delta t}$$

Recidivism rates may vary not only over time but also among firms’ characteristics. In the present study, the objective is to draw inferences about the influence of environmental sanction charges on the hazard of recidivism.

One possible statistical model is Cox ’s (1972) proportional hazards model where sanction charges (here denoted by *z*) affects the hazard of recidivism in a multiplicative manner according to

3 $$\lambda (t|{\mathbf{z}})=\lambda _{0}(t)\exp \left( z\beta \right).$$

Here, $\lambda _{0}(t)$ is an unspecified base-line function of time and $\beta$ is an unknown parameter representing the effect of sanction *z*. The factor $exp(z\beta )$ describes the hazard of recidivism for an individual firm with sanction *z* relative to that of a standard (with *z* = 0 ). Details on estimation and tests on $\beta$ may be found in Cox (1975). Standard statistical software like R, SAS, SPSS, STATA first transform the above model (3) into a linear model:

4 $$\ln \left[ \lambda (t|z)\right] =\ln \left[ \lambda _{0}(t)\right] +z\beta,$$

and provide estimates of $\beta$ together with their standard errors, 95% confidence intervals, as well as estimates of the corresponding relative hazards, $\exp \left( z\widehat{\beta }\right)$.

Results from the above model yielded the following estimates (Table 5).

**Table 5:** Results from a univariate proportional hazards model

Sanctions (SEK)	${\widehat{\beta }}_{i}$	se(${\widehat{\beta }}_{i}$)	Wald-stat	* df*	*p* value	Hazard ratio	95% CI for hazard ratio
<5000	0.72	0.065	121.9	1	0.00	2.06	(1.81; 2.34)
5000–10,000	0.49	0.068	51.8	1	0.00	1.63	(1.43; 1.86)
≥10,000	0	–	–	–	–	1	

Thus, the relative hazard of recidivism of the 1st group of firms (those charged <5000 SEK previously) is 2.06. In other words, these firms have a risk of recidivism that is twice (2.06 times) as that of the baseline group of firms (those charged 10,000 SEK or more previously). Moreover, this difference is highly significant as indicated by the *p* value of 0.00. Similarly, the 2nd group of firms (those charged 5000–10,000 SEK for previous offence) have a relative hazard of 1.63 which, in turn, means their risk of recidivism is 1.63 times that of the baseline group of firms. This difference is also highly significant as indicated by the low *p* value of 0.00. In sum, we note that higher sanction charges are associated with reduced risks of recidivism. To examine for possible interactions between the size of sanctions charges and other characteristics of firms we fitted nested hazards models with results presented in Table 6. The table shows that the relative risk vary depending on whether other covariates are included in the model. This variation is consistent with the frequency distribution of recidivism across cross classifications of these covariates as shown in Table 7. We, therefore, present sanctions-profiles of relative risks across motive (Table 8) and motive-profiles of relative risks across sanctions (Table 9).

**Table 6:** Relative risks of recidivism in nested hazards models (* 0.10 < *p* value < 0.05; ** 0.05 ≤ *p* value < 0.01; *** *p* value ≤ 0.01)

Covariate	Level	Model 1	Model 2	Model 3	Model 4
Sanctions (SEK)	<5000	2.06***	1.98***	1.88***	1.43***
	5000–10,000	1.63***	1.62***	1.56***	1.23***
	≥10,000 (Ref)	1	1	1	1
Number of	None	–	0.20***	0.20***	0.23***
Employees	1–9	–	0.18***	0.18***	0.20***
	10–99	–	0.25***	0.25***	0.27***
	100 or more (Ref)	–	1	1	1
Implementing	Municipalities	–	–	1.22**	1.35***
Authority	County Adm. Board	–	–	1.15	1.75 ***
	Other central auth. (Ref)	–	–	1	1
Motiv group	Group 1	–	–	–	0.70**
	Group 2	–	–	–	0.56***
	Group 3	–	–	–	1.57***
	Group 4 (Ref)	–	–	–	1

**Table 7:** Distribution of events (recidivism) and exposure-months across motive and sanctions (see Table 1 for definition of columns)

Motive	Sanctions	Recid.	Exp	Rate	RR
1	<5000	429	43,422	9.88	3.42
	5000–10,000	43	26,578	1.62	0.56
	≥10,000	71	24,593	2.89	1
2	<5000	306	56,673	5.40	1.30
	5000–10,000	158	69,314	2.28	0.55
	≥10,000	86	20,617	4.17	1
3	<5000	1172	72,374	16.19	1.06
	5000–10,000	862	42,637	20.22	1.32
	≥10,000	74	4838	15.30	1
4	<5000	21	947	22.18	6.68
	5000–10,000	17	1374	12.37	3.73
	≥10,000	40	12,038	3.32	1
	Total	3279	375,405	8.73	–

**Table 8:** Sanctions-profiles of Relative Risks across Motive

Sanctions	Motive 1	Motive 2	Motive 3	Motive 4
<5000	3.42	1.30	1.06	6.68
5000–10,000	0.56	0.55	1.32	3.73
≥10,000	1	1	1	1

**Table 9:** Motive-profiles of Relative Risks across sanctions

Motive	Size of sanctions charge (SEK)		
	<5000	5000–10,000	≥10,000
1	0.45	0.13	0.87
2	0.24	0.69	1.26
3	0.73	1.63	4.61
4	1	1	1

### Accelerated failure-time models for the duration of compliance

In the proportional hazards models (3) the explanatory variables (sanctions charges) act multiplicatively on the baseline hazard so that their effect is to increase or decrease the hazard relative to $\lambda _{0}(t)$. Another class of models, known as accelerated failure-time models which are closer to ordinary linear regression, specifies the covariates to act multiplicatively on event time itself (or linearly on log-failure time) rather than on the hazard function.

Let $T_{0}$ be the time (duration) to recidivism associated with a firm in the baseline (first group) corresponding to zero values for the covariates $(z=0)$, then the accelerated failure time model specifies that if for $z\ne 0$, the event time (duration) to recidivism would be:

5 $$T=T_{0}exp(z\beta)$$

or equivalently, that

6 $$\ln \left( T\right) =\ln \left( T_{0}\right) +z\beta$$

where, as before, *T* is the vector of failure times, ${\mathbf{z}}$ is a vector of covariates or independent variables, $\varvec{\beta }$ is a vector of unknown regression parameters.

Since covariates alter, by a scale factor, the rate at which an individual traverses the time axis, (5) is referred to as the accelerated failure time model. Thus, in accelerated failure-life models the explanatory variables act multiplicatively on time to the event so that their effect is to accelerate or decelerate time to failure relative to $T_{0}$.

One point that is worth noting at this stage is that the parameterizations in (3) and (5) are different. A positive coefficient in (3) implies an increased hazard (shorter duration) while in (5) it implies longer duration (decreased hazard) relative to that of the baseline (where covariates assume the value of zero).

The model in (6) is a linear model with $ln\left( T_{0}\right)$ playing the role of an error term with an underlying baseline distribution. Usually, an intercept term $\alpha$ and a scale parameter $\delta$ are allowed in the model to give

7 $$ln\left( T\right) =\alpha +z\beta +\delta ln\left( T_{0}\right)$$

or more explicitly as

8 $$ln\left( T\right) =z\beta ^{*}+\delta \epsilon$$

where $\beta ^{*}=\left( \alpha \,\, \beta \right)$ and a more conventional notation, $\epsilon$, is used for the random error term. The distribution of the random error term can be taken from a class of distributions that includes the extreme-value, normal, and logistic distributions, and, by using a log transformation, exponential, Weibull, lognormal, log-logistic and gamma distributions. In general, the distribution may depend on additional shape parameter *k* to give (Stacey 1962) a generalized gamma model:

9 $$f(k,\epsilon )=\frac{1}{\Gamma (k)}\exp \left[ k\epsilon -\exp (\epsilon ) \right] ,\quad-\infty<z\beta ^{*}<\infty;\quad-\infty<\epsilon <\infty;\quad \delta ,k>0.$$

Further extensions by Prentice (1974) and Farewell and Prentice (1977) have led to an extended generalized gamma (EGG) distribution with shape parameter $q=k^{- {\frac{1}{2}} }$. Such an EGG distribution is the distribution of *T* when the error term in (8) has the following density function:

10 $$f(q,\epsilon )=\left\{ \begin{array}{ll} \frac{\left| q\right| }{\Gamma (q^{-2})}(q^{-2})^{q^{-2}}\exp \left\{ q^{-2}\left( q\epsilon -\exp (q\epsilon )\right) \right\} ,&\quad q\ne 0 \\ \frac{1}{\sqrt{2\pi }}\exp (-\epsilon ^{2}/2),&{\quad}q=0 \end{array} \right.$$

The EGG distribution reduces to the standard normal distribution for $\epsilon$ when the shape parameter *q* is equal to zero. Accordingly, *T* will have a log-normal distribution. When the shape parameter *q* equals 1, (10) reduces to

11 $$f(q,\epsilon )=\exp \left\{ \epsilon -\exp (\epsilon )\right\} ,{\quad}-\infty<\epsilon <\infty$$

which is the standard (type 1) extreme-value distribution. As $ln\left( T\right)$ is a linear function of $\epsilon$, it has the same (extreme-value) distribution as $\epsilon$. Hence $T=exp(z\beta ^{*}+\delta \epsilon )$ will have a Weibull distribution. If $q=1$ and $\delta =1$, then *T* has the exponential distribution as a special case of the Weibull distribution. The case of $q=-1$ corresponds to extreme maximum-value distribution for *lnT*. This, in turn, corresponds to reciprocal-Weibull distribution for *T*. The case of $\delta =1$ and $q>0$ is also of interest.

Farewell and Prentice (1977) argue that this gives the ordinary gamma distribution for *T* though, in accordance with Bergström and Edin (1992) and Bergström et al. (1994); Bergstrom et al. (1997), this does not hold in our case. Consequently, we shall label this special case ($\delta =1$, $q>0$) the ’gamma’ distribution.

Thus, many common distributions for *T* are included as special cases of the EGG model and this makes it easier to choose among competing alternative models using standard likelihood ratio tests. For more details on estimation and previous applications of the model, see Addison and Portugal (1987, 1992), Bergström and Edin (1992), Bergström et al. (1994); Bergstrom et al. (1997), and Ghilagaber (2005) among others.

Application of this model on our data set gave the following results (Table 10).

**Table 10:** Effects of Sanctions Charges on log-duration to first recidivism under various models (*** *p* value ≤ 0.01)

Covariate	EGG	Rec. Weib.	Lognormal	Weibull	‘Gamma’	Exponential	Loglogistic
Intercept	5.64	4.74	5.65	6.12	10.35	5.43	5.58
Scale parameter ($\delta$)	2.56	3.10	2.55	1.60	1	1	1.41
Shape parameter (*q*)	−0.02	−1	0		19.69	1	–
$\hbox{MSA}<5000$ SEK	−1.10	−0.94	−1.10	−1.22	−1.87	−0.94	−1.15
MSA 5000–10,000 SEK	−0.87	−0.88	−0.87	−0.81	2.21	−0.57	−0.84
MSA ≥10,000 SEK	0.00	0.00	0.00	0.00	0.00	0.00	0.00
−2LogLik	21,409	21,493	21,410	21,511	22,217	22,619	21,484
Diff (Chi-square)	–	84***	1	102***	808***	1210***	–

The baseline categories were firms with previous sanction charges of 10,000 SEK or more. The estimated coefficients represent effects of the other two groups relative the corresponding baseline level (where covariates assume the value of zero) on duration in compliance (time until recidivism).

According to the table, firms in the 1st and 2nd group (those with previous charges less than 10,000 SEK) have shorter compliance durations than those in the 3rd group (those with previous charges of 10,000 SEK or more). The results are consistent across all duration models with regard to the direction of effects though they may vary in terms of strength.

Given the varying strengths of effects across the models, it is natural to ask on which model inferences should be based. The fact that the common distributions like the Weibull and lognormal are nested within the more comprehensive EGG model makes it simple to test the relative merits of the special cases using likelihood ratio tests.

Statistics corresponding to various tests for special cases of the EGG model (10) are presented in the last row of Table 10. These are used to test whether the corresponding special-case model is adequate relative to the more comprehensive EGG model. The results show that reciprocal Weibull, Weibull, ’gamma’ and exponential models are rejected in favor of the more general EGG model. On the contrary, the log-normal model is adequate enough compared to the EGG model ($$\chi ^{2}=$$ $$1<3.84)$$. This is also supported by the estimated value of the shape parameter under the EGG model. The estimates of the shape and scale parameters, as reported in Table 10, are $$-0.02$$ and 2.56, respectively. The estimated shape parameter is, thus, closer to the assertions of the log-normal (in which the shape parameter is fixed to 0 with a free scale parameter) than to any of the values asserted by the other distributions (-1 for reciprocal Weibull and 1 for Weibull). As a result, one can also note that the estimates of the covariates effects in the log-normal model and the EGG model are much closer while those in the other models differ from the estimates of the EGG model. In other words, we have a statistical justification to base our inference on the results from the log-normal model (in the case where only sanctions charges is included as covariate).

When we include all covariates, however, none of the special cases performs as good as the general EGG model and, hence, we prefer to keep the more comprehensive model and draw our conclusions based of estimates from that model. Results from nested EGG models are shown in Table 11.

**Table 11:** Effects of Sanctions Charges on log-duration to first recidivism in nested EGG models (* 0.10 < *p* value < 0.05; ** 0.05 ≤ *p* value < 0.01; *** *p* value ≤ 0.01)

Covariate	Levels	Model 1	Model 2	Model 3	Model 4
Sanctions (SEK)	<5000	−1.10***	−0.90***	−0.80***	−0.39***
	5000–10,000	−0.87***	−0.61***	−0.56***	−0.17
	≥10,000 (Ref)	0.00	0.00	0.00	0.00
No. Employees	None		2.71***	2.71***	2.35***
	1–9		2.95***	2.94***	2.62***
	10–99		2.46***	2.44***	2.20***
	100 or more (Ref)		0.00	0.00	0.00
Authority	Municipalities			−0.35***	−0.46***
	County Adm. Board			−0.42**	−1.01***
	Other Central Auth (Ref)			0.00	0.00
Motive	Cause 1				0.48***
	Cause 2				0.67***
	Cause 3				−0.72***
	Cause 4 (Ref)				0.00

Thus, if $T_{3}$ is the time (duration) to recidivism associated with a firm in the baseline (third group) the event time to recidivism for the 1st group of firms is given by

$${\widehat{T}}_{1}=T_{3}*\exp \left( {\widehat{\beta }}_{1}\right) =T_{3}*\exp \left( -1.10\right) =0.33*T_{3}$$

while that of the 2nd group of firms is given by

$${\widehat{T}}_{2}=T_{3}*\exp \left( {\widehat{\beta }}_{2}\right) =T_{3}*\exp \left( -0.87\right) =0.42*T_{3}$$

Using the results where we control for the other three covariates, we have,

$${\widehat{T}}_{1}= T_{3}*\exp \left( {\widehat{\beta }}_{1}\right) =T_{3}*\exp \left( -0.39\right) =0.68*T_{3}\quad {\widehat{T}}_{2}= T_{3}*\exp \left( {\widehat{\beta }}_{2}\right) =T_{3}*\exp \left( -0.17\right) =0.84*T_{3}$$

A randomly selected firm from the 1st group (with previous sanction of less than 5000 SEK) needs 32% shorter time to commit recidivism compared with a firm selected from the 3rd group (with previous sanction of 10,000 SEK or more). Further, this difference is statistically significant at 5% significance level. On the other hand, a firm randomly selected from the 2nd group (with previous sanction 5000–10,000 SEK) needs about 16% shorter time to commit recidivism compared to 3rd group (with previous sanction of 10,000 SEK or more) but this difference is not statistically significant at 5% level of significance.

## Summary, concluding remarks, and suggestions for future work

### Summary and concluding remarks

In the present study we have analyzed data on environmental recidivism among about 9000 Swedish firms which were fined with environmental sanction charges sometime between January 2002 and December 2012. A firm enters into the study at the date of decision of first known sanction charge and is followed up until it commits a re-offense or the study ends in December 2012, whichever comes first.

We analyzed the data using various statistical methods ranging from the very standard nonparametric comparison of two survival curves to an advanced family of flexible parametric duration models for the association between compliance time and background covariates.

Our empirical results from all the above analytical methods show that penalty (sanctions charges), indeed, has a strong deterring effect on recidivism in the sense that firms which experience higher sanctions charges tend to commit recidivism at much lower rate than those fined with lower sanctions for previous offenses. We also found that the strength of the detering effect depends on whether or not we account for other potential correlates of recidivism.

### Limitations of the study and recommendations for further study

This study has identified appropriate analytic methods to examine the association between a firm’s characteristics (such as size of previous sanction charges) and its risk of committing re-offens. However, apart from the questionable value of recidivism as a measure of effectiveness, the data set used in this study is not without limitations and such limitations should be borne in mind when attempting to draw conclusions from the analyses.

As described before, a firm enters the study at the date of the first known (not necessarily the first ever) decision. The firm is then followed up until the date of the next decision (if it has committed recidivism within the follow up period) or the end of the observation period, December 2012 (if the firm was still complying). The date of decision is different from the date of when the violation was committed and the (strong) assumption made here is the time between date of violation and the date of decision is uniform across the firms. An alternative approach can be to use discrete-time hazard modelling where the events and exposures are agregated over appropriate duration-intervals.

Moreover, the unit of time used in this study is month. However, it is not certain that firms are inspected (or decisions on offenses are made) on a monthly basis. The use of months in the present study may be justified by necessity or convenience—as we have a relatively long follow-up period of 11 years (the longest observed exposure time was, however, 120 months).

Further, the study in this paper focuses on time to first recidivism and we have deleted data referring to more than one recidivism. Including such data in future studies may help examine if inspectors punish multiple offenders significantly more harshly than one-time offenders and if this, in turn, is effective in reducing further recidivism.

The study is based on firms that have been charged with sanctions within the study period and, thus, we have no clue on the behavior of those firms that were not charged within the period (say those charged before January 2002). Further, we have no information on how far back the first offence was committed which, in turn, indicates left censoring. This may affect the estimates as firm’s propensity to re-offend may depend on how far back the first offence was committed.

The results also indicate interaction between the size of sanction charges and the motive for violation. One may suspect that firms that commit relatively ’minor’ offenses are charged with lower charges which, in turn, has less impact on deterring re-offense. This implies that the size of sanction charges may in itself be explained by the motive (type of environmental violation). Examining and adjusting for such endogeneity of the size of sanctions charges requires more advanced multiprocess modelling which can be an area for future investigation.

Finally, the effect of sanctions charges on compliance may vary over time. It may, for instance, have a strong dettering effect immediately after the charges were imposed but its effect may decline in the long term. Thus, modelling approaches that allow effects of covariates to change over time (Gamerman 1991; Wagner 2011; Munezero 2016) may be used to investigate if effects of sanctions charges change over time.

## References

[CR1] Addison JT, Portugal P (1987). On the distributional shape of unemployment duration. Rev. Econ. Stat..

[CR2] Addison JT, Portugal P (1992). The distributional shape of unemployment duration—a reply. Rev. Econ. Stat..

[CR4] Bergström R, Edin PA (1992). Time aggregation and the distributional shape of unemployement duration. J. Appl. Econom..

[CR5] Bergström R, Engwall L, Wallerstedt E (1994). Organizational foundations and closures in a regulated environment: Swedish commercial banks 1831–1990. Scand. J. Manag..

[CR6] Bergstrom R, Engvall L, Wallerstedt E (1997). The importance of flexible hazard functions in the analysis of organizational survival data—experiences from a cohort of Swedish commercial banks. Qual. Quant..

[CR7] Cox DR (1972). Regression models and life-tables (with discussion). J. R. Stat. Soc..

[CR8] Cox DR (1975). Partial likelihood. Biometrika.

[CR9] Farewell VT, Prentice RL (1977). A study of distributional shape in life testing. Technometrics.

[CR10] Gamerman D (1991). Dynamic Bayesian models for survival data. J. R. Stat. Soc. Ser. C (Appl. Stat.).

[CR11] Ghilagaber G (2005). The extended generalized Gamma model and its special cases: applications to modeling marriage durations. Qual. Quant..

[CR12] Herzing, M., Jacobsson, A.: Efficient environmental inspections and enforcement. Report 6713, Swedish Environmental Protection Agency (2016). http://www.naturvardsverket.se

[CR13] International Network for Environmental Compliance and Enforcement, INECE (2008), Performance Measurement Guidance for Compliance and Enforcement Practioners, 2nd edn. INECE Expert Working Group on Enforcement and Compliance Indicators (2008)

[CR14] Munezero, P.: A particle filter for dynamic survival models. Department of Statistics, Stockholm University (2016)

[CR15] Prentice RL (1974). A log-gamma model and its maximum likelihood estimation. Biometrika.

[CR16] Stacey EW (1962). A generalization of the gamma distribution. Ann. Math. Stat..

[CR17] U.S. Environmental Protection Agency—Office of Enforcement and Compliance Assurance: Re-evaluation of the use of recidivism rate measures for EPA’s Civil Enforcement Program. Report to Office of Management and Budget (OMB), 24 June (2008)

[CR18] Wagner H (2011). Bayesian estimation and stochastic model specification search for dynamic survival models. Stat. Comput..

